# Acquired convergence of hormone signaling in breast cancer: ER and PR transition from functionally distinct in normal breast to predictors of metastatic disease

**DOI:** 10.18632/oncotarget.2354

**Published:** 2014-08-16

**Authors:** Heidi N. Hilton, Tram B. Doan, J. Dinny Graham, Samantha R. Oakes, Audrey Silvestri, Nicole Santucci, Silke Kantimm, Lily I. Huschtscha, Christopher J. Ormandy, John W. Funder, Evan R. Simpson, Elizabeth S. Kuczek, Peter J. Leedman, Wayne D. Tilley, Peter J. Fuller, George E. O. Muscat, Christine L. Clarke

**Affiliations:** ^1^ Westmead Millennium Institute, Sydney Medical School – Westmead, University of Sydney, NSW, Australia; ^2^ Cancer Research Program and The Kinghorn Cancer Centre, Garvan Institute of Medical Research, Darlinghurst, NSW, Australia; ^3^ St Vincent's Clinical School, St Vincent's Hospital and University of New South Wales, Darlinghurst NSW, Australia; ^4^ Children's Medical Research Institute, Westmead, New South Wales, Australia; ^5^ MIMR-PHI Institute, Clayton, Victoria, Australia; ^6^ Sydney Medical School, University of Sydney, Westmead, NSW, Australia; ^7^ Laboratory for Cancer Medicine, Centre for Medical Research, Western Australian Institute for Medical Research and School of Medicine and Pharmacology, the University of Western Australia, Perth, Western Australia, Australia; ^8^ Dame Roma Mitchell Cancer Research Laboratories, Discipline of Medicine, Hanson Institute, University of Adelaide, Adelaide, South Australia, Australia; ^9^ Institute for Molecular Bioscience, University of Queensland, St. Lucia, Queensland, Australia

**Keywords:** Estrogen receptor, progesterone receptor, breast cancer, human breast

## Abstract

Cumulative exposure to estrogen (E) and progesterone (P) over the menstrual cycle significantly influences the risk of developing breast cancer. Despite the dogma that PR in the breast merely serves as a marker of an active estrogen receptor (ER), and as an inhibitor of the proliferative actions of E, it is now clear that in the breast P increases proliferation independently of E action. We show here that the progesterone receptor (PR) and ER are expressed in different epithelial populations, and target non-overlapping pathways in the normal human breast. In breast cancer, PR becomes highly correlated with ER, and this convergence is associated with signaling pathways predictive of disease metastasis. These data challenge the established paradigm that ER and PR function co-operatively in normal breast, and have significant implications not only for our understanding of normal breast biology, but also for diagnosis, prognosis and/or treatment options in breast cancer patients.

## INTRODUCTION

Cumulative exposure to estrogen (E) and progesterone (P) throughout a woman's reproductive life significantly influences the overall risk of developing breast cancer [1], and the evidence that ovarian hormones are major drivers of breast cancer risk is irrefutable [2, 3]. Ovariectomy decreases breast cancer risk, while early menarche, late menopause, and late first full-term pregnancy are associated with an increase in breast cancer risk [4]. Similarly, increased breast cancer risk associated with synthetic progestins in exogenous formulations, such as oral contraceptives and hormone replacement therapy (HRT), further highlights the role of ovarian hormones in breast cancer [5, 6], yet the respective roles of E and P in the breast remain undefined.

Development of the normal breast is driven by the endocrine environment, with breast proliferation occurring in puberty [7], during the menstrual cycle [8, 9], and in pregnancy. In animals, E and P have distinct activities in mammary development, with E regulating ductal elongation [10] and P regulating side-branching and lobular development [11]. Expression of the nuclear receptors, estrogen receptor (ER) and progesterone receptor (PR), through which E and P exert their effects, supports the view that these hormones have distinct roles in normal human breast [8, 9]. ER is cyclical over the menstrual cycle, expressed in the follicular phase of the menstrual cycle but virtually absent in the luteal phase and pregnancy [12], when PR is expressed [13]. The proportion of ER+ cells significantly increases with age to a plateau at menopause [14], whereas this same age-related change is not seen for PR. There is also increasing evidence that full-term pregnancy induces long-term gene expression changes in the breast [15], with parous individuals displaying lower expression of *ER and PR* than nulliparous subjects [16-18].

Despite the complexity of E and P action in the breast, and the heterogeneity of ER and PR in the gland, it has long been believed that ER and PR are co-expressed in the same cells, and that PR merely serves as a marker of an active ER, and as an inhibitor of the proliferative actions of E. This view is challenged by evidence that P increases normal breast proliferation independently of E action, and that PR is expressed independently of E stimulation. Moreover, normal breast progenitor cells do not show the same pattern of ER and PR expression, supporting distinct roles for E and P in breast epithelial development [19, 20].

Although there is a paucity of data on the roles of E and P in normal breast, it has long been known that over 60% of breast cancers contain both ER and PR. The presence of ER and PR is the single most important predictive marker for positive endocrine responsiveness [21] yet molecular profiling has identified subgroups within ER+PR+ tumors with widely different prognoses, for example the Luminal B subgroup of ER+PR+ tumors with high proliferation and poor prognosis [22]. Consequently, expression of ER and PR are important but not definitive predictors of prognosis, and indicators of prognosis that incorporate both the expression and functional significance of these steroid receptors are urgently needed. In this study, we have examined a range of human cohorts and used *in vivo*, *in vitro* and *in silico* approaches to identify distinct pathways associated with ER and PR in normal breast, and the convergence of their action in breast cancer, to derive ER-PR focussed prognostic tools to identify different prognosis groups within breast cancers with otherwise good prognosis.

## RESULTS

### Expression of ER and PR is non-overlapping in normal human breast and mouse mammary gland

**Figure 1:**
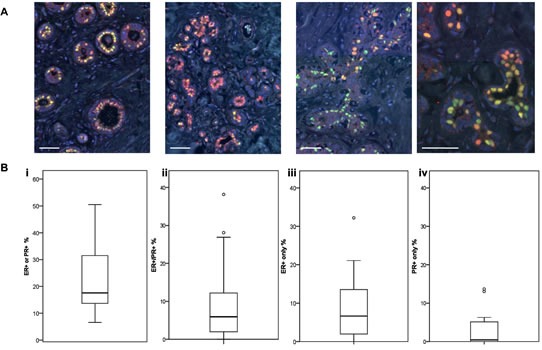
ER and PR expression in normal human breast A Representative IF images of normal breast tissue sections from 4 individual donors stained for ER (red) or PR (green). Scale bars represent 50 μm. B Boxplots (displaying the minimum, first quartile, median, third quartile, maximum and outliers) of the proportions of (i) ER+ or PR+ cells (ii) ER+/PR+ co-expressing cells, (iii) ER+ only cells, and (iv) PR+ only cells in a series of normal human breast tissue samples (n = 22).

Individual ER and PR expression in the normal breast is known to be markedly heterogeneous, but there are few data that have specifically determined whether ER and PR are expressed in the same cells. In a cohort of normal human breast samples (22 independent samples from 20 patients), we revealed extensive heterogeneity in the combined expression of these steroid hormone receptors (HR) in HR+ cells, both within the same breast, and between individual samples (Figure 1A and Supp Fig 1A). Expression of a single HR (ER or PR) was more common than co-expression of ER and PR (Figure 1B). In this sample cohort, a median of 5.9% (range 0-38%) of epithelial cells counted overall co-expressed ER and PR, while 10.1% (range 2.4-32.2%) expressed only one HR. Although the single HR+ cells more frequently expressed ER (median 6.6%; range 0-32%) than PR (median 0.4%; range 0-14%), there were several individual samples which contained more PR+ only cells than ER+ only cells (Supp Fig 1A), evidence that the lower levels of PR positivity were not due to an underestimate of PR expression. Furthermore, the lack of overlap in ER and PR co-expression was clearly evident when sections were stained for only one HR (Supp Fig 1B). There was no correlation between the proportion of HR positivity and age (Supp Fig 1C).

**Figure 2:**
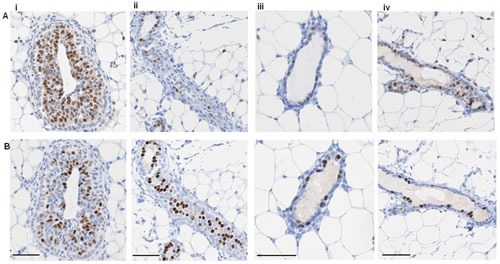
ER and PR expression in mouse mammary gland Representative IPX images of adjacent sections of mouse mammary gland showing (A) ER and (B) PR expression at (i) 5 week virgin, (ii) estrous, (iii) diestrous, and (iv) proestrous. Scale bars represent 50 μm.

Similar to human breast tissue, ER and PR showed distinct expression patterns in the mouse mammary epithelium at various stages throughout the estrous cycle (Figure 2). Circulating levels of E and P rise and fall during the murine estrous cycle, which results in fluctuating activation of ER and PR. This in turn is followed by changes in levels of gene transcription, which affect not only the cells expressing ER and/or PR, but also neighbouring cells via paracrine signalling [2, 23, 24]. The levels of E peak at late proestrous (pre-ovulation) and fall throughout estrous, whereas P levels peak during diestrous [24]. In post-pubertal elongating mammary glands taken from mice at 5 wks age, ER expression was widely expressed by the cells of the proliferating terminal end buds and the luminal epithelium (Figure 2Ai), in contrast PR expression is less widely distributed. Thus in the elongating mammary gland there are both steroid receptor negative and positive cells, and a population of cells that express ER, but have low or no expression of PR. These observations reinforce the importance of E (but not P) for mammary ductal elongation, illustrated by mammary gland reconstitution experiments using ER^-/-^ and PR^-/-^ mammary epithelium showing that ER, but not PR, is necessary for this stage of mammary development [25, 26].

Different patterns of steroid receptor expression were observed in the adult mammary gland (Figure 2ii - iv). In contrast to the elongating mammary gland, ER and PR expression was restricted to a subset of luminal epithelial cells with fewer cells expressing ER. At diestrous, when P levels are high, elevated PR expression was seen (Figure 2Biii). When E levels surge pre-ovulation, so did the expression of ER, with PR levels remaining high (Figure 2iv). In adjacent sections taken from adult mammary glands, we observed that there were both steroid receptor negative and positive cells, plus a proportion of luminal epithelial cells that expressed PR with low or no expression of ER. These observations are consistent with previous investigations [27] and fit with the indispensable role of P-stimulated mammary epithelial cell proliferation during branching morphogenesis [25, 28]. Hence the distinct actions of E and P, acting on their cognate receptors in the mammary epithelium, support the notion that the mouse mammary gland contains distinct cellular populations that respond to systemic hormonal cues in a context-dependent manner at different stages of development. Therefore, in contrast to the general acceptance that HRs are always co-expressed, we have demonstrated here that in both the human breast and mouse mammary gland the expression of ER and PR is highly heterogeneous and largely non-overlapping.

### ER and PR transcripts are enriched in functionally distinct cell subsets in the normal human breast

**Figure 3:**
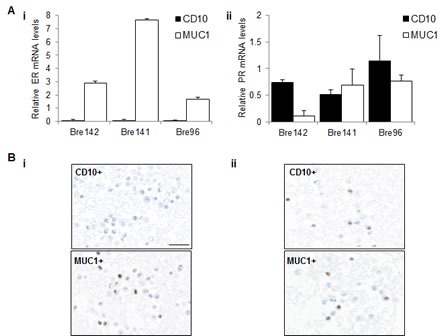
ER and PR expression in luminal and basal cell subsets **A** Relative mRNA levels of ER (i) and PR (ii) were normalised to TBP in basal CD10+ and luminal MUC1+ subpopulations. Graphs represent the mean + SE in independent experiments using tissue from 3 different patients. **B** Representative images showing IPX staining for (i) ER and (ii) PR in sorted CD10+ and MUC1+ fractions. Scale bar represents 25 μm.

To examine the presence of ER and PR in luminal and basal epithelial cell subpopulations, we isolated these subsets from normal breast samples. ER transcripts were highly enriched in each luminal cell fraction, in contrast with PR transcripts, which were enriched in the basal and bipotent progenitor fractions (Figure 3A and Supp Fig 2) [20]. ER protein expression was only detected in the luminal subpopulation (Figure 3Bi), while PR protein was markedly heterogeneous between individuals (Figure 3Aii), and detected in both subpopulations (Figure 3Bii), recapitulating the transcript data. As we [29] and others [30-33] have shown, CD10+ cells display enhanced colony forming ability with a higher proportion of colonies displaying a bipotent progenitor phenotype. This suggests that segregation of ER and PR transcripts in fractionated cell subsets based on different lineage markers may reflect functional differences in the normal breast. Thus, this demonstration of enrichment of ER and PR (transcripts and protein) in distinct cell compartments in an *in vitro* 3D culture model of normal breast tissue supports the IF staining of breast tissue sections *in vivo* depicted in Figure 1.

### Increasing co-expression of ER and PR in pre-invasive breast lesions and in breast tumors are associated with lineage composition and proliferation

**Figure 4:**
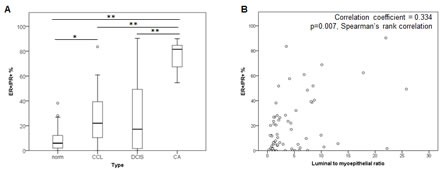
ER and PR expression in a cohort of normal, pre-invasive and breast tumor samples **A** Boxplots of the proportions of ER+/PR+ co-expressing cells across a spectrum of normal (norm; n=22), CCL (n=22), DCIS (n=22) and invasive breast cancer (CA; n=7) samples. ** = p<0.001, * = p<0.01. **B** Scatter plot of distribution of luminal to myoepithelial ratio and percentage of ER+/PR+ co-expressing cells.

We measured ER and PR in pre-invasive lesions (columnar cell lesions (CCL; n=22), DCIS samples; n=22) and invasive breast tumor samples (n=7). Compared with normal breast tissue, the pre-invasive lesions showed higher numbers and a wider range in the proportion of cells co-expressing ER and PR, ranging from 0% to ~85% (Figure 4A and Supp Fig 3A). The tumor samples also contained significantly higher numbers of ER+/PR+ co-expressing cells, compared with both normal breast tissue and pre-invasive samples. There was no consistent change in the proportion of single ER+ or PR+ cells across the sample cohort, although there were much higher numbers of single ER+ cells overall, compared with single PR+ cells (Supp Fig 3B and 3C). These data show that ER and PR expression become more highly correlated in breast tumor samples, than in normal breast.

Cancers show progressively fewer myoepithelial cells relative to luminal cells as they progress through premalignant to malignant stages [34]. We compared luminal to myoepithelial cell ratio and proportion of ER+/PR+ cells in this cohort and found an increased proportion of ER+/PR+ cells in cancers where myoepithelial cells were lower (correlation coefficient = 0.334, p=0.007, Spearmans Rank correlation; Figure 4B). There was a significant correlation between lineage composition and proportion of cells expressing only ER (correlation coefficient = 0.509, p<0.0001, Spearmans Rank correlation, not shown), but no correlation with cells expressing only PR (correlation coefficient = -0.169, p=0.183, Spearmans Rank correlation, not shown). The expression of ER was also associated with proliferation (as measured by the proportion of Ki67+ cells [34]), as reflected by the significant correlation between proliferation and the proportion of cells expressing only ER (correlation coefficient = 0.269, p=0.03, Spearmans Rank correlation). These data raise the possibility of a role for E in pre-invasive lesions, and suggest that E may drive increased proliferation in luminal cells within these lesion types.

### ER and PR are functionally non-overlapping in normal human breast

**Figure 5:**
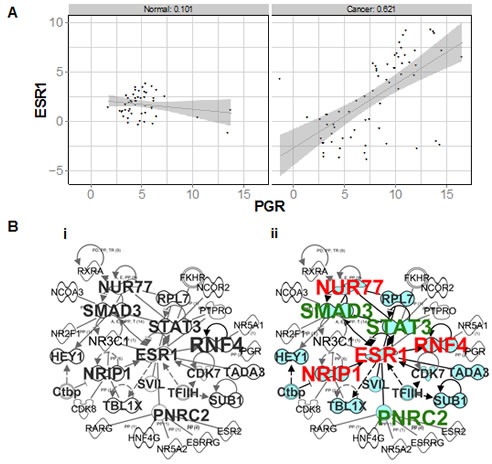
ER and PR transcripts and correlated networks in an independent cohort of normal and breast cancer samples **A** Scatter plot of ΔCt values obtained for ER (ESR1) and PR (PGR) in normal breast vs cancer samples profiled on TLDAs. **B** Merged ER and PR correlated networks in (i) normal, and (ii) breast cancers. Genes which also correlate with ER are shaded in blue. Also indicated are genes which are activated (red) or inhibited (green) by E.

The non-overlapping expression of ER and PR in normal breast was validated in independent cohorts (normal breast n=50; breast cancer n=66), where we had previously measured the transcript expression of ER, PR and the other members of the nuclear receptor (NR; n=48) gene family, and all known NR co-regulators (CoRs; n=238) [35]. This showed that ER and PR transcript levels were not correlated in normal breast (left panel, Figure 5A), confirming the immunohistochemical data. In further support, ontology analysis of genes regulated by P in the normal breast in 3D culture [19], and by E in normal breast xenografts in nude mice [36], revealed that E and P regulated non-overlapping functional pathways (Table 1). Whereas E regulated transcripts were involved in extracellular signalling, P regulated processes were associated with cell growth (RNA processing, cell cycle), consistent with its demonstrated stimulation of proliferation in the breast. In contrast to these non-overlapping pathways, there were only 3 broad categories which contained genes regulated by both E and P, and these had relatively low enrichment scores (Table 1); the individual functions of E and P in the normal human breast are thus distinct. To further explore the functional overlap of ER and PR in normal breast, we identified all NR and/or CoRs that correlated with ER or PR in the normal breast cohort (n=50). There were 35 genes in total that correlated with ER and 32 with PR, but none correlating with both ER and PR (Supp Fig 4A), supporting the findings in Table 1.

**Table 1:** Ontology analysis of E-regulated functional pathways compared with P-regulated, and combined E/P-regulated pathways in normal human breast

	
E Regulation Summary function	Enrichment score
Chemokine signalling	3.77
Extracellular signalling Hormone response	2.89 2.85
Extracellular metabolism	2.34
**P regulation Summary function**	**Enrichment score**
RNA processing	9.77
Macromolecule assembly	3.29
Cell cycle	3.27
Mitochondrial organisation Cell cycle regulation	2.97 2.40
Serine peptidase inhibition	2.21
ECM binding	2.03
**Combined E and P regulation Summary Function**	**Enrichment score**
Wound response Cell adhesion Hormone response	2.68 2.40 2.12

### ER and PR become correlated in breast cancer and converge on common pathways involved in cytokine signaling, cell cycle control, and pluripotency

In contrast with normal breast, ER and PR transcripts became highly correlated in breast cancers (right panel, Figure 5A). To identify whether this was associated with functional overlap, we identified 120 NR and CoR genes that correlated with either ER or PR in breast cancers (n=66). Whereas in normal breast ER and PR were correlated with non-overlapping sets of NRs and CoRs, we identified genes (n=22) that were correlated with both receptors only in breast cancer. Moreover, Ingenuity network analysis of NR and CoR genes that were highly correlated with PR in breast cancers, but not normal breast, revealed an ER (ESR1)-centred network of genes (Figure 5B). Upstream regulator analysis revealed that E was the most common shared upstream regulator of PR correlated genes, showing that PR-correlated genes become E-regulated in cancer. NUR77, NRIP1 and RNF4 signalling is activated by E (shown in red), whereas SMAD3, STAT3 and PNRC2 regulated functions are inhibited by E (shown in green). NUR77 is frequently elevated in cancers [37] and NRIP1 promotes mitogenic signalling in the developing mammary gland [38], while SMAD3 and STAT3 are known to be fundamental mediators of growth factor and cytokine signalling. Furthermore, from our dataset of PR genomic interactions in breast cancer cells [39], we have identified PR binding sites close to all six of these PR correlated genes (Supp Table 1), suggesting that they may be directly PR regulated.

These data, discovered in robustly validated cohorts of human tissues, demonstrate that PR signalling converges with that of ER in breast cancers, in an interplay that does not exist in normal tissue. Moreover, to determine the functional significance of this converged E and P signalling, Ingenuity overlapping pathways analysis of this network and its direct connections revealed significant over-representation of pathways associated with cell cycle control, cytokine signalling and maintenance of pluripotent state (Supp Fig 4B). This demonstrates that genes correlated with PR and ER in breast cancer are involved in pathways crucial to cancer cell development and function. Therefore, we have shown in an additional sample cohort using alternative methodologies that ER and PR transcripts do not correlate in normal breast tissue, and become more highly correlated in breast tumors. This is supported by the demonstration that ER and PR each correlate with non-overlapping transcriptional networks in normal breast, but that ER and PR networks overlap in breast cancer.

### Expression of genes highly correlated with both ER and PR in breast cancers are associated with distinct clinical outcomes

**Figure 6:**
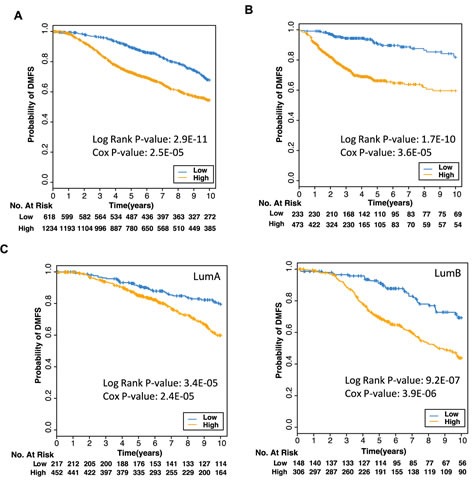
Expression of 18 genes predicts poorer prognosis Kaplan Meier plot of probability of survival where patient samples from the **(A)** METABRIC breast cancer microarrays, or **(B)** from three different datasets (GSE25055, GSE25065, GSE7390), were stratified into low or high risk groups based on low (blue) or high (orange) expression of the 18 genes found to be correlated with both ER (ESR1) and PR (PGR) expression in cancers. **C** Kaplan Meier plots of patient samples from Luminal A tumors (left panel) or Luminal B tumors (right panel) in the METABRIC dataset were stratified into low or high risk groups.

Using the METABRIC breast cancer microarray dataset of 1853 samples [40], we identified 18 genes that were highly correlated with both ER and PR (Spearman Rank Correlation >= 0.5) in both the discovery and validation cohorts (Supp Table 2). Although the function of a large number of these genes is not known, there were a number of metabolic markers, for example, NAT1 [41] and ABAT [42]. Interestingly, 10 of the 18 genes contained both ER and PR binding regions within 50 kb of their transcription start sites (indicated in Supp Table 2), and most of these genes contained ER and PR overlapping binding sites (indicated in Supp Table 3). To determine the clinical significance of these 18 genes, we tested the association between this gene signature and patient survival in the METABRIC dataset [40]. Sample risk scores calculated based on the combined weighted expression of the 18 genes in the signature showed significant correlation with distant metastasis-free survival, with higher risk scores correlating with poorer prognosis (Figure 6A; Cox *p* value = 1.23x10^-11^). Importantly, this persisted even when accounting for node status and ER/PR expression, indicating that this signature can identify patients with risk of earlier metastasis, independent of ER and PR status. The association between expression of the 18 gene signature and patient prognosis was also validated in three independent breast cancer microarray datasets (GSE25055, GSE25065 and GSE7390; Figure 6B).

Finally, we looked at the association between expression of the 18 gene signature and distant metastasis-free survival within particular tumor subtypes. The levels of expression of this 18 gene signature were able to identify differences in prognosis both within the Luminal A subtype, and to a higher degree within the Luminal B intrinsic subtypes (Figure 6C). Furthermore, lower risk scores calculated based on the expression of the 18 gene signature identified patients with a better prognosis only in a unique subset of these tumors expressing high levels of PR but low levels of ER (Supp Fig 5). Interestingly, the gene signature also identified patients with a better prognosis in ER- tumors (data not shown), although this was less significant. Therefore, by measuring the ER-PR correlated 18 gene signature in a number of highly curated datasets, we have identified a prognostic signature which identifies patients with ER+PR+ breast cancer who are at risk of earlier metastasis, particularly in Luminal B cases, and can thus predict poorer outcome independent of ER or PR status.

## DISCUSSION

This study demonstrates that in the normal human breast, the expression of ER and PR do not correlate and that their functions are largely non-overlapping. This expression pattern of ER and PR is also seen in a cohort of pre-invasive lesions. In contrast, we show that ER and PR become highly correlated in breast cancer, and that their actions converge. Finally, we have identified a novel potential prognostic gene signature which identifies patients with ER+PR+ breast cancer at risk of earlier metastasis. Importantly, these data were obtained using a combination of both human and mouse samples, transcript and protein data, multiple sample cohorts, and *in vitro*, *in vivo* and *in silico* methods.

The expression of ER and PR is highly heterogeneous in the normal breast, both inter- and intra-individually, and there is large variation in intensity of expression of each HR, which results in a rainbow-like appearance in stained sections. Aside from natural inter-individual variation, these variable proportions of cells expressing only one HR, or both, is likely to be due to differences in menstrual cycle phase, exogenous hormone use and/or parity, information which was not available to us. The sequence of antibodies applied is of critical importance to accurately reflect the heterogeneous expression (Supp Fig 6), but heterogeneity is still evident when mirror sections are stained with only one HR (Supp Fig 1B). Identification of ER and PR transcripts in different cell compartments based on various progenitor or mature cell markers suggests that both HRs can be expressed individually, and at multiple stages of the epithelial cell hierarchy. Indeed, we have previously shown that ER, and to a greater extent PR, can be expressed in basal cells, functionally and phenotypically consistent with being progenitor cells [20]. Intriguingly, we observed these HR+ progenitor cells expressed only ER or PR, but not both, and so it is tempting to speculate that, in the normal breast, a subset of the cells which express only one HR, are progenitor cells.

Heterogeneous HR expression is also evident in pre-invasive breast lesions. The CCLs and DCIS lesions are associated with significantly higher risks for tumor formation, and the relative risk increases progressively according to the histological subtype of the lesion. While variable risk ranges have been reported, the relative risk factors are accepted to be ~1.5 for CCL and ~10 for DCIS [43, 44]. It is important to note the wide distribution of ER+/PR+ co-expressing cells in the pre-invasive lesions, and it would be interesting to determine whether those with higher proportions of co-expressing cells are indicative of convergence of ER and PR. Such lesions may be more pre-disposed to development of steroid receptor-positive tumor formation, in which case HR co-expression may serve as an additional marker of prognostic significance. Moreover, it is tempting to speculate that lesions expressing more ER, or PR, or co-expressing both, may give rise to tumors of different phenotypes.

The correlation between the proportion of luminal cells, and the proportion of cells expressing either ER only, or both ER and PR, highlights a potential role for ER in lineage re-modeling in carcinogenesis. As an increase in proportion of luminal cells correlates with increasing severity of the lesion [34], this supports the notion that ER expression, and ER/PR co-expression, are also more highly correlated with increasing severity. The lack of correlation between increased luminal cell numbers with increased PR+ only cells however, raises the possibility that ER-/PR+ tumors may be more likely to resemble basal tumors. Indeed, a recent study reported that the majority of ER-/PR+ tumors examined were in fact basal-like tumors [45]. This would also be consistent with the possibility that these tumors may arise from PR+ bipotent progenitor cells.

In ER+PR+ breast tumors, the proportion of cells expressing these HRs is commonly higher than in the normal breast [46], thus the increased proportion of ER+/PR+ co-expressing cells might reflect a higher probability of each HR being expressed in the same cell. Alternatively, more luminal lesions may arise from ER+/PR+ co-expressing cells. In the present study we did not distinguish individual steroid receptor isoforms. Previous studies have shown, however, that in the earlier stages of carcinogenesis increased levels of ERα are expressed, with a clear dissociation between ER expression and proliferation [1]. Furthermore, the ERβ isoform is preferentially lost in some cancers, whereas its introduction slows the growth of breast cancer cells [47], supporting the notion that dysregulation of HR expression either contribute to, or accompany breast tumorigenesis.

The lack of overlap in the normal breast between the regulatory pathways correlated with either ER or PR is striking, underlining the independent action of each hormone in the normal breast. In contrast, this study shows that E and P have converging roles in breast cancer, specifically on pathways associated with cell cycle control, cytokine signaling and stem cell regulation, all commonly up-regulated during tumorigenesis. Moreover, E stimulates human breast cancer stem cells (CSCs) [48], as do P and progestins [29, 49, 50]; there are also data that E reduces stem cell frequency in breast cancer [51]. As breast tumors are postulated to arise from carcinogenic transformation of stem/progenitor cells [52], it will be critical to identify whether E and P signaling converges in distinct cell types within the epithelial hierarchy during tumorigenesis, potentially giving rise to different breast cancer subtypes.

Finally, identification of pathways common to both ER and PR in breast cancer has yielded an 18 gene signature associated with higher risk of early metastasis. High expression of a number of these genes has also been identified in previous studies to be linked to a poorer prognosis, for example, SIAH2 [53], and NAT1 and SCUBE2 [54]. Interestingly, SCUBE2 is the only gene in common between the genomic assays, Oncotype DX and MammaPrint. The 18 gene signature identified in this study was rich in metabolic markers, and it has long been known that tumor cells display higher rates of glycolysis, believed to promote unconstrained proliferation [55]. Thus this gene signature may identify tumors which have a significant growth advantage and thus likely to have a poorer outcome. It must also be noted that the functions of 10 of the 18 genes remain unknown, and identification of function of these genes has the potential to discover novel pathways with a role in driving increased breast cancer risk.

There are common misconceptions regarding the role of E and P in the breast, which largely derive from what we understand of their functions in the uterus, where proliferation occurs prior to ovulation and post-ovulatory secretion of P inhibits proliferation. Moreover, current thinking supports the idea that PR expression is mediated by ER activation, again reflecting uterine biology. The findings of this study clearly demonstrate that the current understanding of E and P action in the normal human breast, and expression of their receptors, has important consequences, and urgently needs to be re-evaluated. For example, such a high degree of heterogeneity in HR expression within the population would mean that administration of exogenous hormone formulations such as HRT or the oral contraceptive pill would have vastly different effects in different people. Moreover, E and P within these hormone formulations would largely not be targeting the same cells. The findings also support the hypothesis that PR acquires the capacity to interact with the ER pathway in breast cancer, and that their combined action impacts on breast cancer development and progression, particularly where endogenous hormone cyclicity is disturbed, such as in peri-menopause and during HRT exposure. As HRT is one of the most frequently administered pharmaceuticals world-wide, delineating the mechanisms of an altered interaction between PR and ER in cancer is crucial to unravelling the mechanisms that underlie the role of HRT progestins in breast cancer, and would lead to potential strategies for continued development and administration of hormone formulations. Moreover, delineating the mechanisms of an altered interaction between PR and ER in cancer may identify potential indicators of breast cancer risk, prognosis and/or treatment options.

As ER and PR status are routinely assessed in all newly diagnosed invasive breast tumours and in breast cancer recurrences, as well as their expression being the most important predictive marker for prognosis and response to endocrine therapy, these findings have critical implications for decisions made regarding diagnosis, prognosis and/or treatment options in breast cancer patients. Moreover, as the action of these hormones underpins both normal development and breast tumorigenesis, the data presented here suggest that the current concepts of how these hormones interact requires re-evaluation.

## MATERIALS AND METHODS

### Tissue samples

Normal (n = 18) and ductal carcinoma *in* situ (DCIS; n = 20) breast tissue samples, and 2 tissue microarrays (TMAs; consisting of normal breast tissue, columnar cell lesions, ductal carcinoma *in situ* samples and tumor samples) which expressed ER and/or PR (>5% positive of epithelial cells counted) were obtained from the Australian Breast Cancer Tissue Bank (http://www.abctb.org.au). Data obtained from breast tissue samples from the cohort described in Muscat *et al*., 2013 [35] were also used. Normal human breast tissue samples obtained from reduction mammoplasty specimens were obtained with informed consent from donors, and the use of all samples was approved by Human Research Ethics Committees of the Sydney West Area Health Service, the University of Sydney, and the Sydney Adventist Hospital, New South Wales, Australia. All mouse work was approved and conducted under the guidelines of the Garvan/St. Vincent's Animal Ethics Committee. Nulliparous wild type FVB/n mice were collected at 5 wks estrous (n=3), 12 wks estrous (n=3), 12 wks diestrous (n=2) and 12 wks pro-estrous (n=1). Estrous staging was performed using cytological examination of DiffQuik stained vaginal smears according to manufacturer's instructions (Fisher Scientific).

### Fluorescence-activated cell sorter (FACS) analysis

Normal human mammary epithelial cells (MECs) were isolated from reduction mammoplasty specimens, and the resulting epithelial organoids were grown in matrix-embedded culture, as described previously [19, 56]. After 9 days of culture, the acini were harvested, dissociated [20] and stained with specific cell markers. For MUC1/CD10 cell sorting experiments, single primary MECs from 3D cultures were stained with anti-MUC1 conjugated with fluorescein isothiocyanate (FITC; BD Biosciences) and anti-CD10 conjugated with phycoerythrin (PE; BD Biosciences). Single primary MECs were also sorted into subfractions on the basis of EpCAM, CD49f, CD10, MUC1, Thy1 and CD133, as described in [20]. For all flow cytometry experiments, forward scatter and side scatter plots were used to account for debris and aggregated cells. Single stained samples were used as compensation controls, and control samples consisted of unstained cells and/or IgG antibodies directly conjugated with FITC and PE (BD Biosciences). Cells were sorted on a FACSAria III cell sorter (BD Biosciences) in the Flow Cytometry Centre at the Westmead Millennium Institute. Analysis was performed using FACS Diva software (v6.1.2, BD Biosciences).

### RNA preparation and qPCR

Sorted primary MECs were maintained at 4°C and collected by centrifugation. RNA was harvested with the Absolutely RNA Microprep Kit (Stratagene) according to the manufacturer's instructions, and reverse transcribed with the High-Capacity cDNA Reverse Transcription kit (Applied Biosystems). The cDNA was amplified by Platinum SYBR Green qPCR Supermix (Life Technologies, Inc.) on a Rotor-gene 6000 real-time cycler (Corbett Research, Australia). The TATA-binding protein (TBP) was used as an internal control gene, and primers used were as described in [20].

### Immunohistochemistry

Matrigel cultures or thrombin clots were fixed, paraffin-embedded, cut into 2-µm sections, and antigens retrieved [57]. Where indicated, sequential sections were cut as mirror sections, to permit staining of opposite, or mirror faces, of the same cells in each section. Immunoperoxidase (IPX) staining was performed as previously published [58]. For dual immunofluorescence (IF) staining on all sample types, antigens were revealed sequentially by incubation of ERα (Novocastra) and PR (AB; Novocastra) primary antibodies. This was followed by detection by a biotinylated goat anti-mouse secondary antibody (Dakocytomation, Glostrup, Denmark) and a streptavidin-conjugated fluorescent label (Alexa 594 or 488; Life Technologies, Inc.). It was critical to apply the ER antibody first, rather than PR, in order to detect each HR accurately, as using the reverse sequence results in underestimation of ER protein expression, and all steroid receptor-positive cells appear predominantly red/orange in colour with a distinct absence of green nuclei, which are clearly present when the PR antibody is applied second (Supp Fig 6). Selected sections were also stained with Ki67 (Dakocytomation, Glostrup, Denmark) primary antibody followed by detection by a biotinylated goat anti-rabbit. All sections were mounted in Prolong Gold antifade reagent containing the nuclear counter-stain DAPI (Life Technologies, Inc.). To ensure antibody specificity, adjacent sections were stained without the primary antibody, using the biotinylated secondary antibody and streptavidin-conjugated fluorescent label only.

For mouse tissue sections, formalin fixed paraffin embedded sections were dewaxed, dehydrated and antigen retrieval performed with pH6 retrieval solution (Dako S1699) in a pressure cooker at 125°C for 30 sec. Endogenous peroxidase was blocked by 3% H2O2 and sections were stained with an antibody to ERα (Santa Cruz MC20 SC-542) or PRA and PRB (Dako A0098) for 30 mins at RT. The sections were then incubated with Envision rabbit HRP secondary (Dako K4002) for 30 min at RT prior to application of 3,3'Diaminobenzidine plus tertiary substrate (Dako K3468) for 10 mins at RT. Sections were counter stained with hematoxylin and slides were imaged with a NanoZoomer (Hamamatsu Photonics, Japan) slide scanner at 400x magnification.

### Quantitation of IF staining

Automated quantitation of normal, pre-invasive and breast tumor samples which had been dual stained for ER and PR was performed using the “cell scoring” module of MetaMorph® software program (version 7.7.5; Molecular Devices). The number of cells counted varied depending on the size of the section or TMA core, with an average of approximately 3000 cells counted per sample. Quantitation of luminal CK18+, myoepithelial p63+ and Ki67+ cells was performed beforehand, as published in [34].

### Statistical methods

Two-sample comparison between subgroups used the Mann-Whitney U test to establish the distribution of the steroid receptor expression in normal, premalignant lesion and tumor groups, and Spearman's rank correlation coefficient used to examine the association of between lineage composition and steroid receptor expression on SPSS software (Version 19, SPSS Inc., Chicago IL).

### TLDA analysis method

The expression correlations of ER and PR transcripts in a cohort of 116 breast tissue samples were profiled on Taqman Low-Density Arrays (TLDA), as carried out in [35]. Briefly, we employed custom designed micro-fluidic TLDAs from ABI, which included probes for 48 nuclear receptors, 238 co-regulators and 16 internal control genes. The 116 breast tissue samples included four groups: ER+ (n=33), ER- (n =33), premenopausal normal (n =30), and postmenopausal normal (n = 20). TLDAs were analysed by relative quantification (ΔCt). The geNorm algorithm implemented in the Integromics StatMiner software package was used to select the most stable house-keeping genes as reference for normalisation. The Ct values of each assayed gene were then normalised against the median of the selected house-keeping genes to obtain the ΔCt values. The pairwise Spearman Rank Correlation Coefficient of ΔCt values was calculated between ER and PR, and between each of the two genes against all other nuclear receptors and co-regulators, which were profiled on the array. Ingenuity Pathway Analysis (Ingenuity® Systems,. www.ingenuity.com). was used to analyse pathway enrichment in particular subgroups of this cohort. To determine whether there were binding sites for ER or PR in the proximity of genes of interest, we interrogated the ChIP-Seq datasets described in Clarke and Graham (2012) (GEO accession GSE31130) [39] and Schmidt *et al* (2010) (ArrayExpress accession E-TABM-828) [59]. ChIP-Seq data were aligned to human genome build hg19 and binding peaks were identified using MACS1.4 [60] with default parameters. A binding site was considered to be near to the gene of interest if it was within 50 kb of the transcription start site.

### Prognostic values of genes highly correlated with both ER and PR in breast cancers

The association between patient survival and expression level of genes highly correlated with both ER and PR in breast cancers were examined in a large publicly available breast cancer microarray dataset of 1853 samples [40]. The normalised expression value as published by the METABRIC study was used to calculate the pairwise Spearman Rank Correlation Coefficient between ER or PR against all other genes profiled on the Illumina HT-12v3 array. Pairwise correlation coefficients were calculated separately for the discovery (EGAD00010000210) and validation (EGAD00010000211) cohort of the METABRIC study. This cohort included 721 Luminal A tumors (of which 669 had clinical follow-up data) and 492 Luminal B tumors (of which 454 had clinical follow-up data) [40]. Genes that correlate with both ER and PR (Spearman Rank Correlation >= 0.5) in both the METABRIC discovery and validation cohorts were retained to be included in the ER-PR gene signature (18 genes). Each patient microarray sample was then assigned a risk score calculated as a weighted average of the expression levels of the 18 ER-PR signature genes using the “sig.score” function from the Genefu R package. Samples were then stratified into 2 risk groups based on the assigned risk scores. The low-risk group comprises samples with risk scores in the bottom tertile, and the high-risk group comprises of samples with risk scores in the top 2 tertiles. Kaplan Meier analysis was performed to test the difference in probability of patient survival of the two risk groups. The association between risk scores calculated based on expression of the 18 ER-PR signature genes with patient survival was also tested by Cox Regression adjusted for Nodal status and ER and PR expression. The significant association between patient survival and expression of genes correlated with both ER and PR in breast cancers was validated in additional independent breast cancer microarray datasets (GSE25055, GSE25065 and GSE7390) profiled on the Affymetrix Human Hg133A arrays. For these Affymetrix microarray datasets, fRMA normalized expression values were downloaded from InSilicoDb. The fRMA normalization algorithm utilizes pre-computed estimates of probe-specific effects and variances obtained from large publicly available databases. These pre-computed values are then used in concert with information from new array(s) for normalization and summarization allowing datasets processed separately to be compared. A risk score was assigned to each microarray sample and the association between the calculated risk scores and patient survival determined as described above.

## Supplementary Material Figures and Tables


