# Supplementary material for: Acquired convergence of hormone signaling in breast cancer: ER and PR transition from functionally distinct in normal breast to predictors of metastatic disease
Source: Oncotarget. 2014 Aug 16;5(18):8651–64. doi: 10.18632/oncotarget.2354 (PMC4226711; doi:10.18632/oncotarget.2354)
Supplement: Supplementary file 1 [file oncotarget-05-8651-s001.pdf]

Acquired convergence of hormone signaling in breast cancer: ER and PR transition from functionally distinct in normal breast to predictors of metastatic disease

Supplementary Material

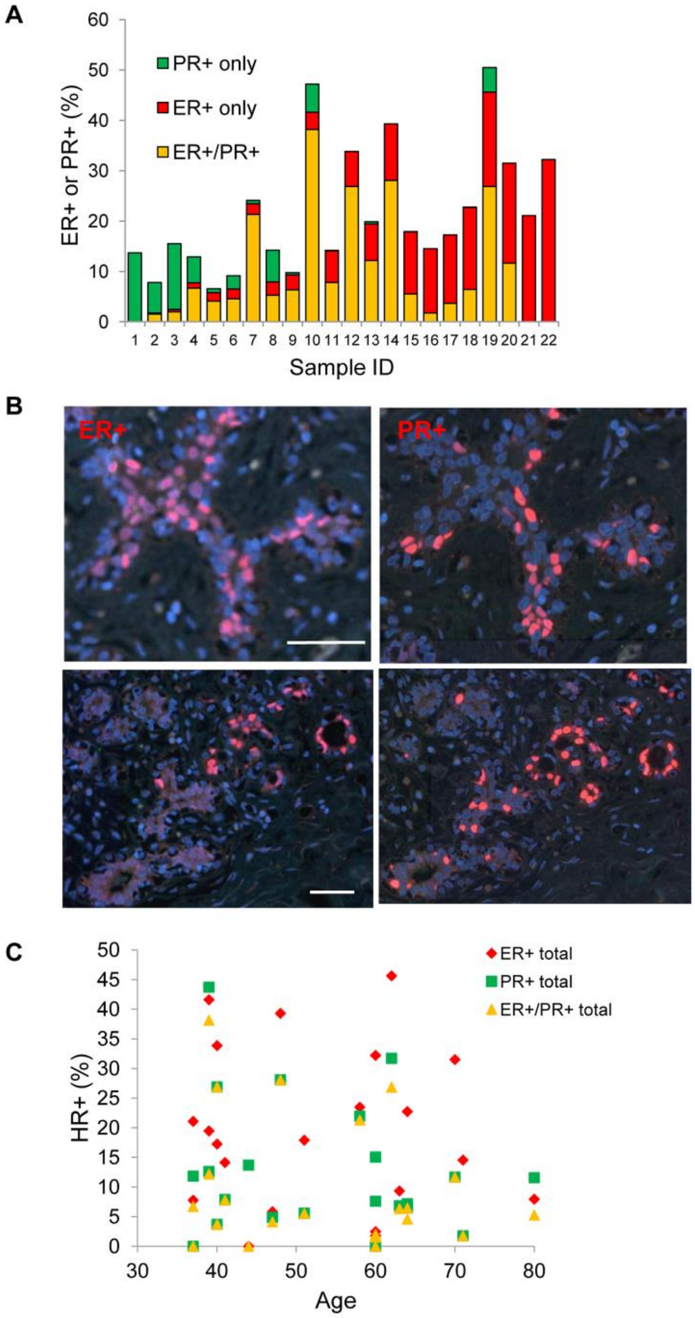

Supplementary Figure 1: A Number of cell nuclei positive for ER only, PR only, or both were quantitated, and expressed as the percentage of total epithelial cell nuclei counted. **B** Mirror sections of normal human breast tissue stained using only ER (left) or only PR (right). Scale bars represent 50 µm. **C** Scatterplot depicting the relationship between hormone receptor status and age.

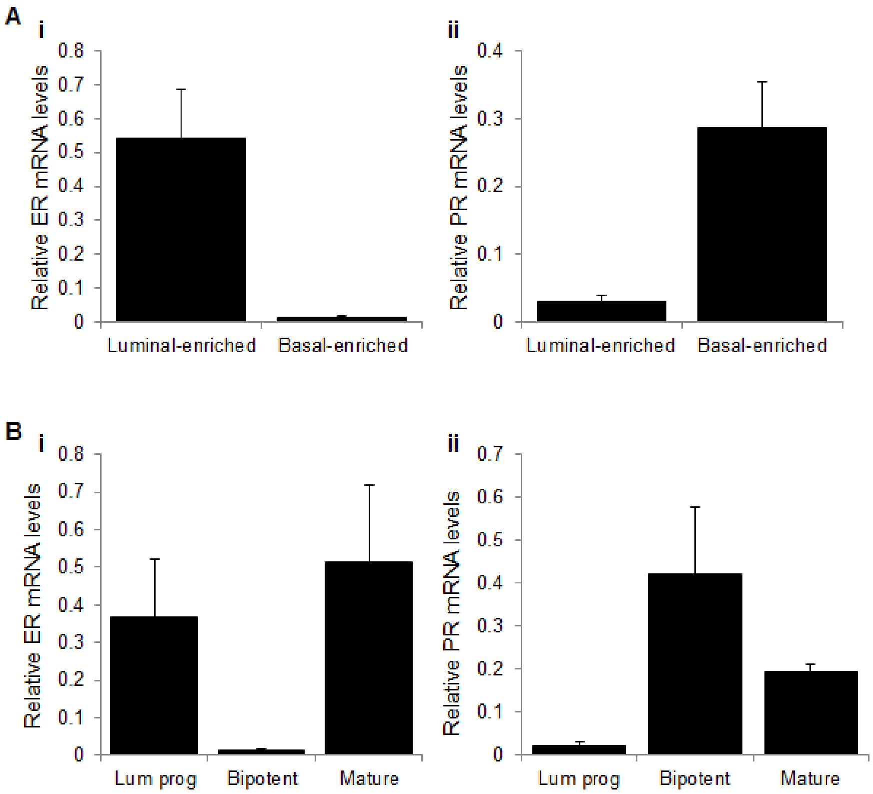

Supplementary Figure 2: A Relative mRNA levels of ER (i) and PR (ii) were normalised to TBP in luminal-enriched and basal-enriched subpopulations sorted on the basis of EpCAM and CD49f markers. **B** Relative mRNA levels of ER (i) and PR (ii) in subpopulations fractionated on the basis of luminal progenitor (CD49f+MUC1+CD133+), bipotent progenitor (CD49f+CD10+Thy1+) and mature (CD49f-) lineage markers. Graphs represent the mean + SE from experiments using tissue from 3 different patients.

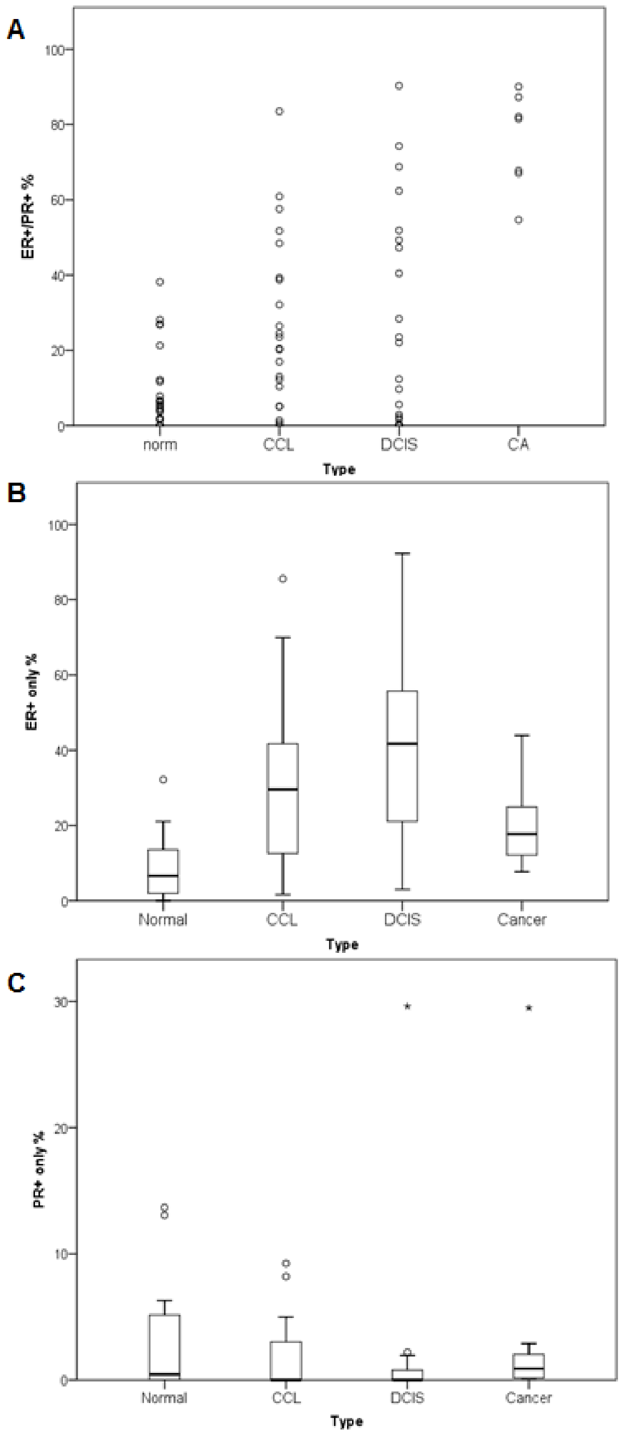

Supplementary Figure 3: A Scatterplot of the proportion of cell nuclei positive for both ER and PR across a spectrum of normal (norm), CCL, DCIS and cancer (CA) samples. **B** and **C**

Boxplots of the proportions of **(B)** ER+ or **(C)** PR+ only expressing cells across the sample cohort.

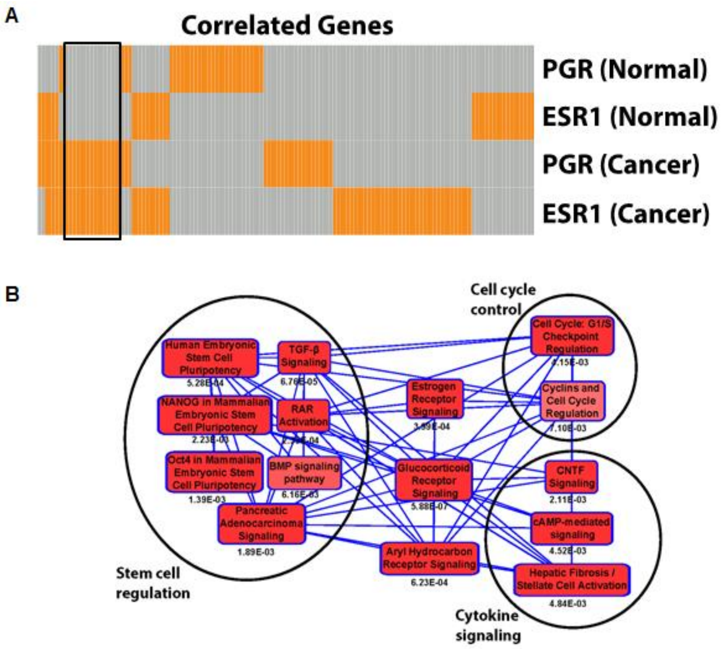

Supplementary Figure 4: A Heatmap representation of the set of 141 genes found to be correlated (Spearman Rank Correlation >= 0.5) with either ER (ESR1) or PR (PGR) in cancer or normal samples based on expression profiling of 48 NRs and 283 CoRs on TLDAs. The box outlines genes which are highly correlated with both ER and PR in cancer, but not in normal breast. **B** Ingenuity overlapping pathways analysis identified over-representation of 3 functional pathways.

.

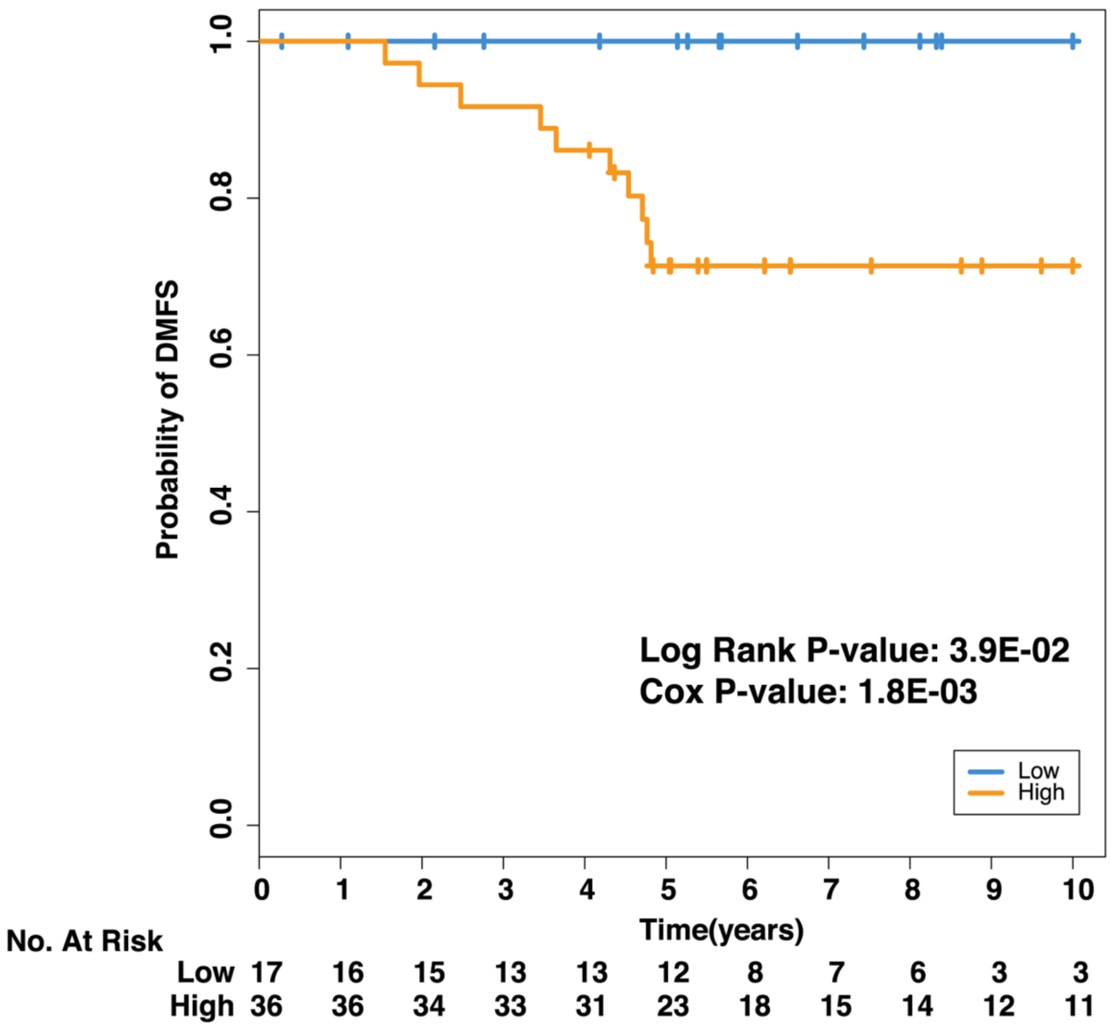

Supplementary Figure 5: Kaplan Meier plot of probability of survival where patient samples from tumors expressing high PR and low ER were stratified into low or high risk groups.

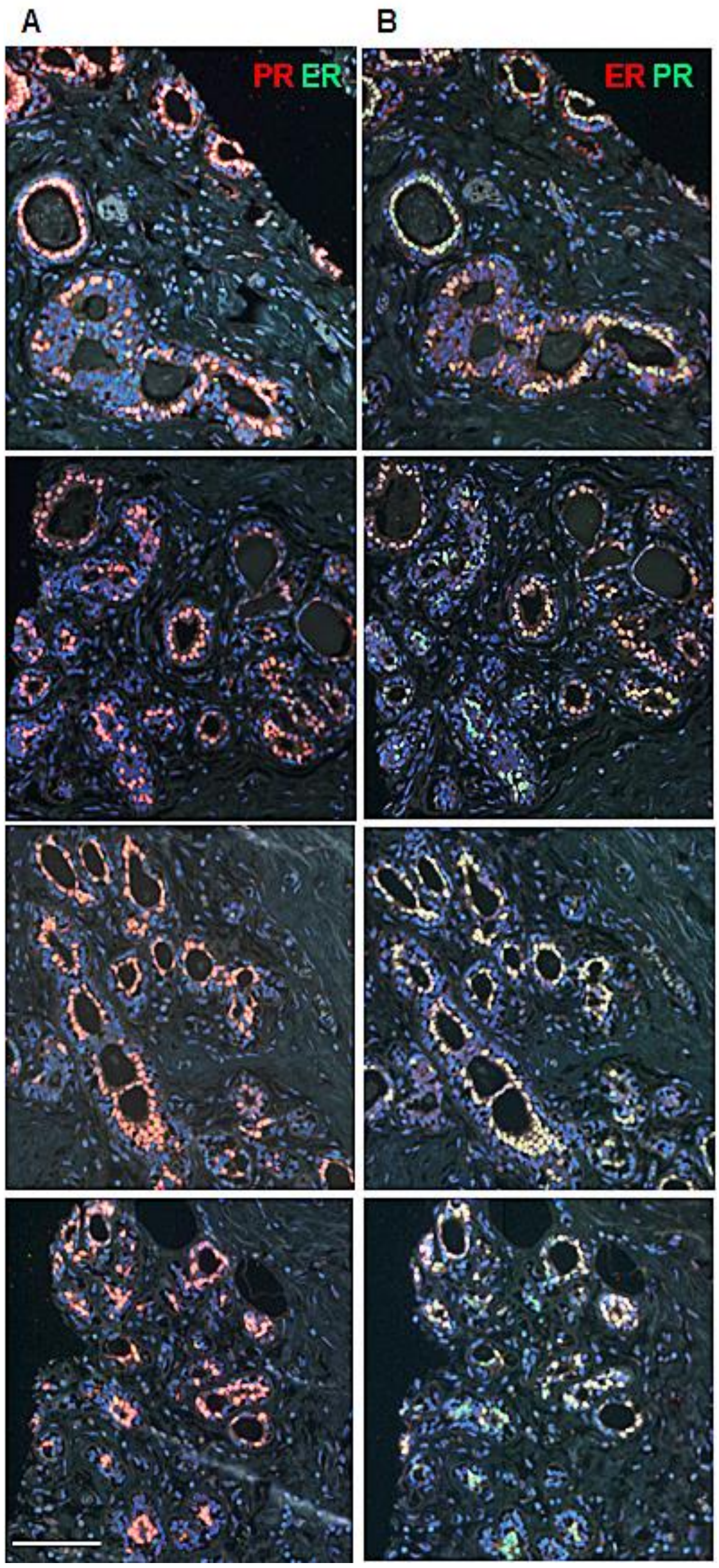

Supplementary Figure 6: Representative images of two sets of mirror sections of normal human breast tissue stained sequentially using **(A)** PR (red) then ER (green), or **(B)** ER (red) then PR (green).

Supplementary Table 1: PR binding sites close to the promoters of PR correlated genes.

Chr	Peak Score	Distance to TSS	Nearest PromoterID	Entrez ID	Nearest Unigene	Gene Name
chr6	64.87	-74105	NM_001122742	2099	Hs.208124	ESR1
chr6	53.66	-56739	NM_001122742	2099	Hs.208124	ESR1
chr6	102.84	-41929	NM_001122742	2099	Hs.208124	ESR1
chr6	547.49	-27437	NM_001122742	2099	Hs.208124	ESR1
chr6	1573.14	-23186	NM_001122742	2099	Hs.208124	ESR1
chr6	89.39	-12955	NM_001122742	2099	Hs.208124	ESR1
chr6	183.12	-10822	NM_001122742	2099	Hs.208124	ESR1
chr6	735.43	-7279	NM_001122742	2099	Hs.208124	ESR1
chr6	591.64	6391	NM_001122742	2099	Hs.208124	ESR1
chr6	54.84	-43624	NM_001122741	2099	Hs.208124	ESR1
chr6	165.26	7329	NM_000125	2099	Hs.208124	ESR1
chr6	242.28	61688	NM_000125	2099	Hs.208124	ESR1
chr6	75.53	86570	NM_000125	2099	Hs.208124	ESR1
chr6	55.62	93549	NM_000125	2099	Hs.208124	ESR1
chr4	105.76	20770	NM_001185009	6047	Hs.740360	RNF4
chr21	384.81	83391	NM_003489	8204	Hs.155017	NRIP1
chr21	109.96	58004	NM_003489	8204	Hs.155017	NRIP1
chr17	114.13	473	NM_213662	6774	Hs.463059	STAT3
chr12	55.71	-6984	NM_001202233	3164	Hs.670088	NR4A1
chr15	382.45	-34524	NM_005902	4088	Hs.727986	SMAD3
chr15	51.32	-2337	NM_005902	4088	Hs.727986	SMAD3
chr15	456.48	-11368	NM_001145104	4088	Hs.727986	SMAD3
chr15	776.97	8758	NM_001145104	4088	Hs.727986	SMAD3
chr15	192.56	19571	NM_001145104	4088	Hs.727986	SMAD3
chr1	105.51	-492	NM_017761	55629	Hs.716935	PNRC2

Supplementary Table 2: Genes highly correlated with both ER and PR in breast cancer samples. Genes indicated with an asterisk contain both ER and PR binding sites within their promoters.

**EntrezGene ID**	**EntrezGene Symbol**	**Gene Name**	**Gene Function** (UniProtKB/Swiss-Prot)
26166	RGS22	Regulator of G-protein signalling 22	Inhibits signal transduction by increasing the GTPase activity of G protein alpha subunits thereby driving them into their inactive GDP-bound form
18	ABAT*	4-aminobutyrate aminotransferase	Catalyzes the conversion of gamma-aminobutyrate and L-beta-aminoisobutyrate to succinate semialdehyde and methylmalonate semialdehyde, respectively
9	NAT1*	N-acetyltransferase 1 (arylamine N-acetyltransferase)	Participates in the detoxification of many hydrazine and arylamine drugs
83450	LRRC48*	leucine rich repeat containing 48	Unknown
26018	LRIG1*	leucine-rich repeats and immunoglobulin-like domains 1	Acts as a feedback negative regulator of signaling by receptor tyrosine kinases
55259	CASC1	cancer susceptibility candidate 1	Unknown
4137	MAPT	microtubule-associated protein tau	Promotes microtubule assembly and stability, and might be involved in the establishment and maintenance of neuronal polarity
1952	CELSR2	cadherin, EGF LAG seven-pass G-type receptor 2	Receptor that may have an important role in cell/cell signaling during nervous system formation
138162	C9orf116*	chromosome 9 open reading frame 116	Unknown
3572	IL6ST*	interleukin 6 signal transducer/gp130	Binds to IL6/IL6R (α chain) complex, resulting in the formation of high-affinity IL6 binding sites, and transduces the signal
80129	CCDC170*	coiled-coil domain containing 170	Unknown
57758	SCUBE2	signal peptide, CUB domain, EGF-like 2	Unknown
203328	SUSD3*	sushi domain containing 3	Unknown
285386	TPRG1*	tumor protein p63 regulated 1	Unknown

256394	SERPINA11	serpin peptidase inhibitor, clade A (α-1 antiproteinase, antitrypsin), member 11	Unknown
6478	SIAH2*	siah E3 ubiquitin protein ligase 2	E3 ubiquitin-protein ligase that mediates ubiquitination and subsequent proteasomal degradation of target proteins
80127	C14orf45	coiled-coil domain containing 176	Unknown
51364	ZMYND10	zinc finger, MYND-type containing 10	Unknown

Supplementary Table 3: Binding sites of ER and PR in 18 gene signature. Genes in red indicate overlapping binding sites of ER and PR.

Entrez Gene ID	*Entrez Gene Symbol*	*PR peak Distance to TSS (kb)*	*ER peak Distance to TSS (kb)*
18	***ABAT***	*-8.6; -1.7; 3.8; 11.6*	*-16.0; -0.4; 1.4; 2.6; 4.4; 6.1; 6.7; 8.5; 11.5; 18.1*
80127	*C14orf45*		
138162	***C9orf116***	*3.4*	*0.1; 3.5*
55259	*CASC1*		
80129	***CCDC170***	*-0.4*	*0.2; 2.1; 42.0*
1952	*CELSR2*		*-11.1; -9.4; -7.7; -3.6; -0.9; 0.7; 1.8; 3.0; 5.7; 13.7*
3572	***IL6ST***	*-44.9; -34.6; -24.7; -8.9; -0.3; 2.4*	*-35.2; -30.6; -18.6; -0.5; 0.08; 33.1*
26018	***LRIG1***	*1.9; 4.1; 8.1; 14.5; 18.2; 19.8; 22.2; 24.7; 31.2; 37.1; 39.9*	*1.9; 16.9; 23.0; 24.8; 26.6; 27.9; 30.8; 37.2; 41.5*
83450	***LRRC48***	*10.8; 15.7; 27.2*	*7.2; 10.9; 12.6*
4137	*MAPT*		
9	***NAT1***	*-41.4; -1.1; -0.1; 9.1*	*9.5; 27.7; 42.3*
26166	*RGS22*		*-17.9*
57758	*SCUBE2*		*-0.4; 0.4*
256394	*SERPINA11*		*0.0; 2.4*
6478	***SIAH2***	*6.7; 18.6; 26.4*	*-23.3; -10.3; -7.3; -6.2; -4.1; -2.7; -0.5; 5.5; 6.7; 11.4; 12.1; 15.0; 15.9; 19.9; 24.9; 26.0*
203328	*SUSD3*	*-9.1; 15.0*	*-39.2; -32.8; -5.0; 1.5; 13.8*
285386	*TPRG1*	*-50.4*	*-44.5; 1.9*
51364	*ZMYND10*		
